# Suppression of P2X3 receptor‐mediated currents by the activation of α_2A_‐adrenergic receptors in rat dorsal root ganglion neurons

**DOI:** 10.1111/cns.13774

**Published:** 2021-12-04

**Authors:** Jia‐Wei Hao, Wen‐Long Qiao, Qing Li, Shuang Wei, Ting‐Ting Liu, Chun‐Yu Qiu, Wang‐Ping Hu

**Affiliations:** ^1^ School of Basic Medical Sciences Hubei University of Science and Technology Hubei China; ^2^ Department of Pharmacology School of Pharmacy Hubei University of Science and Technology Hubei China

**Keywords:** current, dorsal root ganglion neuron, nociceptive behavior, P2X3 receptor, α_2A_ adrenoceptor

## Abstract

**Aims:**

The α_2_‐adrenergic receptor (α_2_‐AR) agonists have been shown to be effective in the treatment of various pain. For example, dexmedetomidine (DEX), a selective α_2A_‐AR agonist, can be used for peripheral analgesia. However, it is not yet fully elucidated for the precise molecular mechanisms. P2X3 receptor is a major receptor processing nociceptive information in primary sensory neurons. Herein, we show that a functional interaction of α_2A_‐ARs and P2X3 receptors in dorsal root ganglia (DRG) neurons could contribute to peripheral analgesia of DEX.

**Methods:**

Electrophysiological recordings were carried out on rat DRG neurons, and nociceptive behavior was quantified in rats.

**Results:**

The activation of α_2A_‐ARs by DEX suppressed P2X3 receptor‐mediated and α,β‐methylene‐ATP (α,β‐meATP)‐evoked inward currents in a concentration‐dependent and voltage‐independent manner. Pre‐application of DEX shifted the α,β‐meATP concentration‐response curve downwards, with a decrease of 50.43 ± 4.75% in the maximal current response of P2X3 receptors to α,β‐meATP in the presence of DEX. Suppression of α,β‐meATP‐evoked currents by DEX was blocked by the α_2A_‐AR antagonist BRL44408 and prevented by intracellular application of the G_i/o_ protein inhibitor pertussis toxin, the adenylate cyclase activator forskolin, and the cAMP analog 8‐Br‐cAMP. DEX also suppressed α,β‐meATP‐evoked action potentials through α_2A_‐ARs in rat DRG neurons. Finally, the activation of peripheral α_2A_‐ARs by DEX had an analgesic effect on the α,β‐meATP‐induced nociception.

**Conclusions:**

These results suggested that activation of α_2A_‐ARs by DEX suppressed P2X3 receptor‐mediated electrophysiological and behavioral activity via a G_i/o_ proteins and cAMP signaling pathway, which was a novel potential mechanism underlying analgesia of peripheral α_2A_‐AR agonists.

## Graphical abstract

The α_2A_‐adrenergic receptors (α_2A_‐ARs) primarily couple the G_i/o_ subtype of G‐protein family, which can inhibit intracellular adenylyl cyclase (AC), resulting in the decrease in cAMP levels and PKA activity. The activation of α_2A_‐ARs by dexmedetomidine (DEX) suppressed P2X3 receptors via the signaling pathway in rat DRG neurons. As a result of DEX treatment, α,β‐methylene‐ATP (α,β‐meATP), a P2X3 receptor agonist, evoked a reduced membrane current and then a decreased burst of action potentials (APs), leading to a relief of α,β‐meATP‐induced pain.

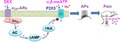

## 1 INTRODUCTION

Noradrenaline is a major monoaminergic neurotransmitter and has many important functions through action on adrenergic receptors, such as α_1_, α_2_, and β receptors.^1^ It has known that noradrenaline is also involved in pain modulation. Among three adrenergic receptors, α_2_‐adrenergic receptors (α_2_‐ARs) play a key role in mediating pain modulatory effects of noradrenaline.^2^, ^3^ The α_2_‐ARs are distributed in the pain signaling pathway, including primary afferents and spinal dorsal horn.^4^, ^5^, ^6^, ^7^ In the spinal cord, norepinephrine released from descending pathways results in a presynaptic inhibition of pain by activating α_2_‐ARs on central terminals of primary afferent nociceptors.^2^ The α_2_‐AR agonists can also mimic the noradrenergic projection of descending pain inhibition.^2^ Dexmedetomidine (DEX), a potent highly selective α_2A_‐AR agonist, has shown potential analgesic effects in animals and humans when administered intrathecally or systemically.^8^, ^9^, ^10^ The α_2_ adrenergic drugs that include DEX and clonidine are approved as analgesic agents in clinical settings.^11^

Systemic DEX analgesia could be blocked by peripheral α_2_‐AR antagonists in neuropathic pain, suggesting a peripheral anti‐nociceptive effect of DEX.^12^, ^13^ The peripheral effect of DEX on nociception is mediated by peripheral α_2_‐ARs, which have been identified in the dorsal root ganglion (DRG). Mechanisms of DEX peripheral analgesia may underlie the modulation of a number of ligand‐gated and voltage‐gated ion channels, which are also expressed in DRG neurons. For example, DEX has been found to inhibit sodium channels through α_2_‐ARs located on DRG and the trigeminal ganglion neurons.^14^, ^15^ DEX also suppresses the activity of TRPV1 via an α_2_‐ARs and cAMP / protein kinase A (PKA) signaling pathway in DRG neurons.^16^

P2X3 receptor is a purinergic ATP receptor. P2X3 homomeric and P2X2/3 heteromer receptors are distributed in DRG neurons and mainly located in a subset of small and medium‐sized nociceptive neurons.^17^, ^18^, ^19^ These peripheral P2X3‐containing receptors contribute to the transmission of nociceptive signaling. For example, blocking P2X3 receptors by antagonists or antisense oligonucleotide can effectively reduce nociception.^20^, ^21^ P2X3 receptor‐mediated currents in DRG neurons and nociceptive behaviors increase after inflammation and nerve injury.^22^, ^23^, ^24^, ^25^ It has shown that P2X3 receptors are regulated by adrenergic signaling. The mRNA assessments indicate both α_1_‐ARs and α_2_‐ARs are expressed in DRG.^3^, ^4^ Noradrenaline potentiates ATP‐evoked currents in DRG neurons by activating PKC via G_q_ protein‐coupled α_1_‐ARs.^26^ Considering the presence of α_2_‐ARs and P2X3 receptors in DRG neurons, it was still unclear whether P2X3 receptors were also modulated by activation of α_2_‐ARs. Herein, we observed that the activation of α_2A_‐ARs by DEX inhibited the electrophysiological activity of P2X3 receptors via an intracellular cAMP signaling pathway in rat DRG neurons. DEX also relieved P2X3 receptor‐mediated nociceptive behaviors in rats by activating peripheral α_2A_‐ARs.

## 2 MATERIALS AND METHODS

### 2.1 Preparation of DRG neurons

All experimental protocols were approved by the animal research ethics committee of Hubei University of Science and Technology. All animal data reporting has followed the ARRIVE 2.0 guidelines (PMID: 32663096). Sprague‐Dawley male rats (5–6 weeks old) were anesthetized and then killed. The DRGs from rats were removed and chopped. The minced ganglia were transferred to a test tube containing Dulbecco's modified Eagle's medium (DMEM) and incubated in a shaking for 25–30 min at 35°C. Incubation solution contained 1.0 mg/ml collagenase, 0.5 mg/ml trypsin, and 0.1 mg/mL IV DNase. Trypsin digestion was terminated by adding 1.25 mg/mL soybean trypsin inhibitor. The cells were cultured for 12–24 hours at 37°C in DMEM containing never growth factor (100 ng/mL) and fetal bovine serum (10%).

### 2.2 Electrophysiological recordings

Electrophysiological experiments were performed as described previously.^27^, ^28^ MultiClamp‐700B amplifier and Digidata‐1550B A/D converter (Axon Instruments, CA, USA) were used for whole‐cell patch clamp recordings. The isolated DRG neurons were transferred to a 35‐mm culture dish and kept in normal external solution for at least 60 min before electrophysiological recordings. The external solution contained the following (in mM): 150 NaCl, 5 KCl, 2 MgCl_2_, 2.5 CaCl_2_, 10 HEPES, and 10 d‐glucose. Its pH and osmolarity was adjusted to 7.4 with NaOH and 330 mOsm/L with sucrose, separately. Recording pipettes were pulled using a Sutter P‐97 puller (Sutter Instruments, CA, USA), and its resistance was in the range of 3–6MΩ. The micropipette solution contained (in mM): 140 KCl, 2 MgCl_2_, 11 EGTA, 10 HEPES, 4 ATP, and 0.3 Na_2_GTP. Its pH and osmolarity was adjusted to 7.2 with KOH and 310 mOsm/L with sucrose, separately. After whole‐cell configuration established, 70–80% series resistance and membrane capacitance current were compensated. The recording currents were sampled at 10 kHz and filtered at 2 kHz. DRG neurons (15–35μm in diameter) are used for electrophysiological recording. The membrane potential of neurons was clamped at −60 mV. Only DRG neuron with a resting membrane potential less than −50 mV was used for current‐clamp recordings.

### 2.3 Drug application

All drugs were obtained from Sigma (St. Louis, MO, USA). The working concentration of drugs was freshly prepared in normal external solution. Each working drug was stored in a series of independent reservoirs and applied by gravity. The distance was ~30 μm between drug exit and recorded neurons. To block G protein and intracellular signal, some antagonists or blockers were dissolved in the internal solution and applied for intracellular dialysis through recording patch pipettes as described previously.^28^, ^29^ To ensure that dialysis drugs are infused into the cell interior, current recording was performed at least 30 minutes after cell membrane rupture.

### 2.4 Nociceptive behavior induced by acetic acid in rats

Male rats were first habituated for 30 minutes in a Plexiglas chamber during the nociceptive behavior experiment. After coding, rats in different groups were intraplantarly pre‐treated with vehicle, different dose (10, 30, and 100 ng in 50 μl) of DEX, or 150 ng BRL44408 + 100 ng DEX, separately. After 10 min, another experimenter injected α,β‐methylene‐ATP (α,β‐meATP, 50 μg in 50 μl) into the ipsilateral hindpaw. In one group, α,β‐meATP was injected into one hindpaw and DEX (100 ng) was injected into contralateral hindpaw, and nociceptive behaviors (ie, number of flinches) were monitored over a 10‐min period starting immediately after the injection of α,β‐meATP. Meanwhile, mechanical allodynia was measured by paw withdrawal threshold (PWT).^30^ PWT of the ipsilateral hind plantar uses a series of von Frey filaments (Stoelting, Wood Dale, IL) at 0.5, 2.5, 5, and 24h after α,β‐meATP injection.

### 2.5 Data analysis

All data were expressed as mean ± SEM. The normality of the data distribution was analyzed by the Shapiro‐Wilk test. If data were normally distributed, the data were statistically compared using Student's t test or analysis of variance (ANOVA), followed by Bonferroni's post hoc test. Nonlinear curve‐fitting program ALLFIT was used for statistical analysis of concentration‐response data.

## 3 RESULTS

### 3.1 DEX inhibits P2X3 receptor‐mediated ATP currents in rat DRG neurons in a concentration‐dependent manner

**FIGURE 1:**
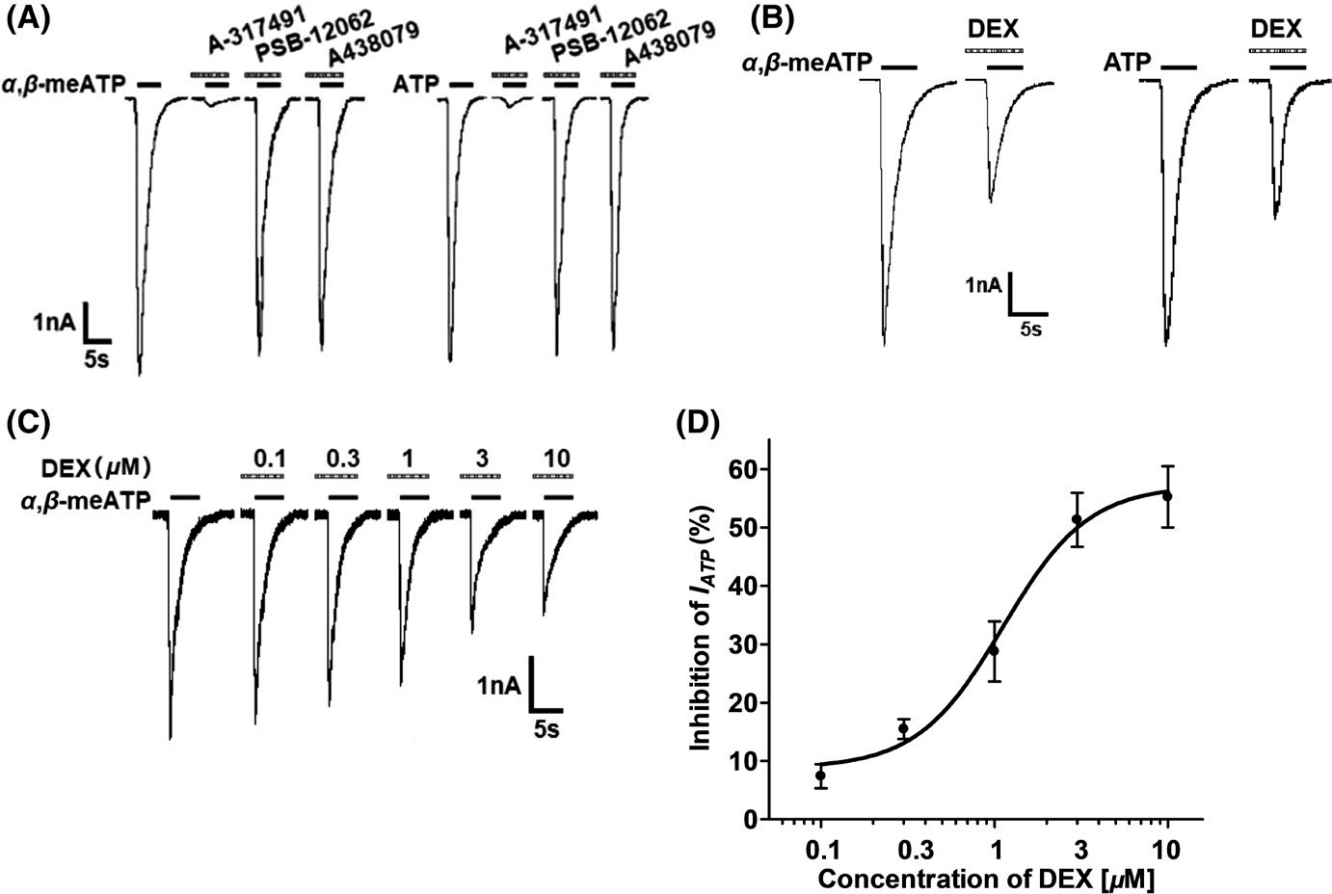
DEX‐induced inhibition of P2X3 receptor‐mediated ATP currents in DRG neurons. (A) Two original currents were evoked by 100 μM α,β‐meATP and 100 μM ATP, separately, in the same DRG cell. These currents were blocked by 300 μM A‐317491 (a specific P2X3 and P2X2/3 receptor antagonist), but not by 10 μM PSB‐12062 (a P2X4 receptor antagonist) and 1 μM A438079 (a potent P2X7 receptor antagonist). Membrane potentials were clamped at −60 mV. (B) Pre‐application of DEX (3 μM for 5 min) to a DRG cell inhibited 100 μM α,β‐meATP‐ and 100 μM ATP‐induced currents similarly. (C) The sequential current traces illustrated that the amplitude of the 100 μM α,β‐meATP‐induced currents progressively decreased after a representative DRG cell was pre‐treated with increasing concentrations of DEX. DEX was pre‐applied to DRG tested for 5min. (D) The graph showed the concentration‐effect curve of DEX on inhibition of 100 μM α,β‐meATP‐induced currents (I_ATP_). The IC_50_ value of the curve was 1.12 ± 0.16 μM. Each point represents the mean ±SEM of 8–10 cells

In the majority of small‐ and medium‐sized (15–40 μm in diameter) DRG cells (66.7%, 6/9), an application of α,β‐methylene‐ATP (α,β‐meATP, 100 μM) or ATP (100 μM) evoked a rapid inward currents (I_ATP_) at a holding potential of −60 mV (Figure 1A). These α,β‐meATP‐ and ATP‐activated currents were blocked by the specifical P2X3 or P2X2/3 receptor antagonist A‐317491(300 μM), but not by the P2X4 receptor antagonist PSB‐12062 (10 μM) and the potent P2X7 receptor antagonist A438079 (1 μM).^21^ Considering that α,β‐meATP is only an activator of P2X3 and P2X1 receptors, we thus concluded that the α,β‐meATP‐activated currents were P2X3 or P2X2/3 receptor‐mediated ATP currents.^31^ The α,β‐meATP‐evoked inward currents were characterized by rapid desensitization with a mean inactivation time constant of 2068 ± 361 ms (n = 8).

In some DRG neurons, we pre‐treated with DEX for 5min prior to the next I_ATP_ recording. As shown in Figure 1B, DEX pretreatment (3 μM) decreased the peak amplitudes of both α,β‐meATP‐ and ATP‐activated currents. Figure 1C shows that the peak amplitudes of 100 μM α,β‐meATP‐activated currents decreased as the concentration of DEX increased from 0.1 μM to 10 μM in a representative DRG cell. Figure 1D shows concentration‐effect curve of DEX on I_ATP_ with an IC_50_ (half‐maximal effective concentration) value of 1.12 ± 0.16 μM. The results suggested that DEX inhibited P2X3 receptor‐mediated ATP currents in a concentration‐dependent manner.

### 3.2 Concentration‐response and current‐voltage relationships for α,β‐meATP in the absence and presence of DEX

**FIGURE 2:**
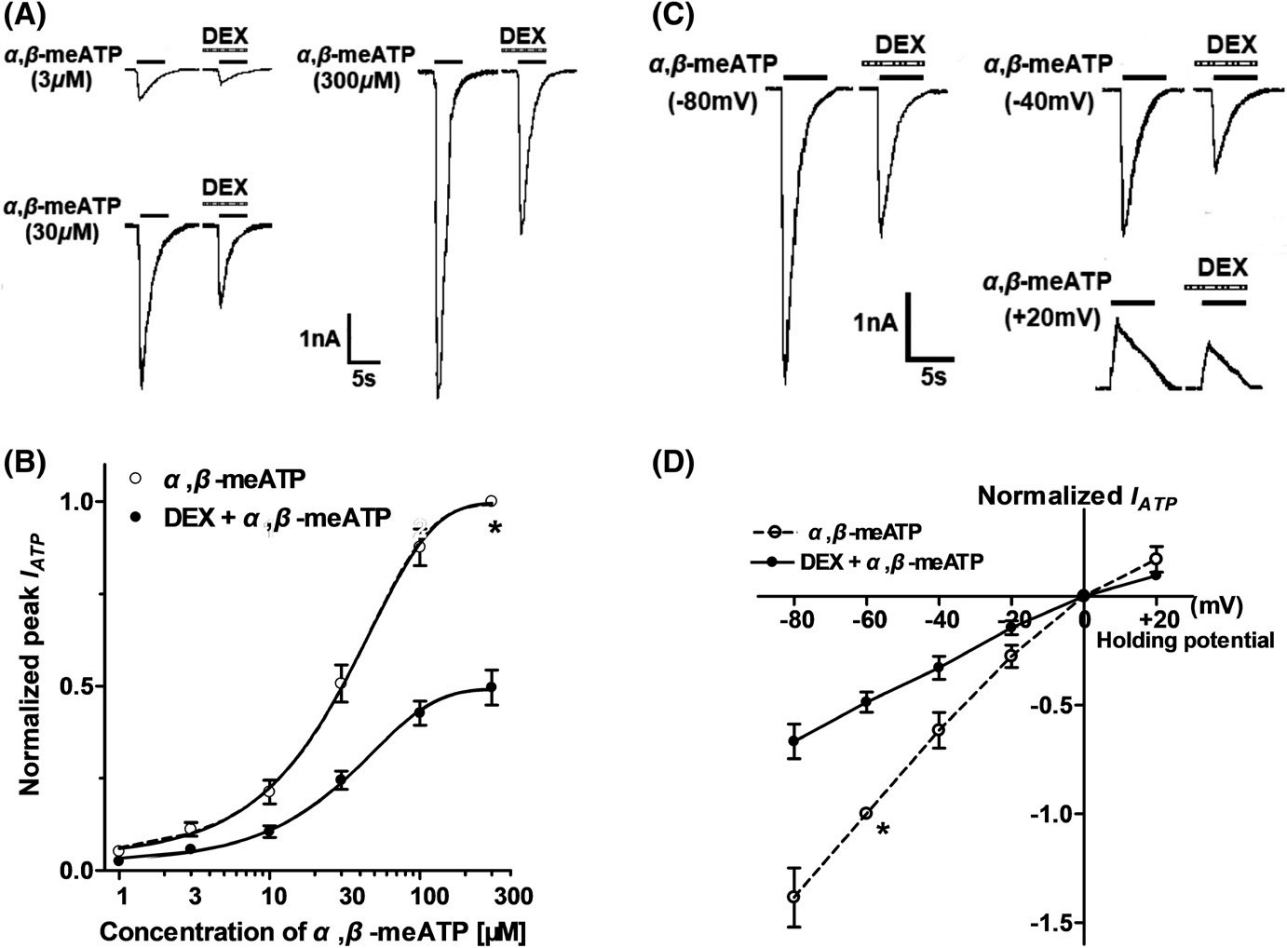
Concentration‐response and current‐voltage (I–V) relationships for α,β‐meATP in the absence and presence of DEX. (A) Sequential currents were evoked by three different concentrations of α,β‐meATP before and after the pre‐application of DEX (3 μM for 5 min). (B) The concentration‐response curves for α,β‐meATP were shifted downwards in the presence of DEX (3 μM). Each point represents the mean ±SEM. of 7–10 cells. All current values from the same cell were normalized to the current response, which was induced by 300 μM α,β‐meATP applied alone in the absence of DEX (marked with asterisk). (C) Sequential currents were evoked by 100 μM α,β‐meATP at three different clamped potentials before and after the pre‐application of DEX (3 μM for 5 min). (D) The I‐V curves for 100 μM α,β‐meATP‐induced currents (I_ATP_) in the absence and presence of DEX (3 μM). All current values from the same cell were normalized to the current response induced by 100 μM α,β‐meATP applied alone at the holding potential of −60 mV (marked with asterisk). Each point represents the mean ±SEM of 7–10 cells. The experiment was carried out using recording pipettes filled with CsCl containing internal solution

We then studied whether the DEX‐induced inhibition depended on the concentration of α,β‐meATP. Concentration‐response curves were plotted through a series of different concentration of α,β‐meATP. Figure 2A shows that three representative ATP currents evoked by α,β‐meATP at 3, 30, and 300 μM were decreased after DEX pretreatment (3 μM) for 5 min. Figure 2B shows that concentration‐response curves for α,β‐meATP were fit with the Hill equation in the absence and presence of DEX (3 μM). We observed that DEX pretreatment shifted downwards the concentration‐response curve for α,β‐meATP. First, maximal current response, which was evoked by 300 μM α,β‐meATP, decreased 50.43 ± 4.75% with DEX (3 μM) pretreatment. Second, the Hill coefficient or slope of curves without and with DEX pretreatment was 1.40 ± 0.26 and 1.32 ± 0.37, respectively, with no significant difference (*p* > 0.1, Bonferroni's post hoc test). Third, DEX pretreatment did not change the EC_50_ of α,β‐meATP for P2X3 receptors, which were 29.45 ± 1.26 μM and 31.03 ± 1.49 μM, respectively, in the absence and presence of DEX (*p* > 0.1, Bonferroni's post hoc test). These results suggested that DEX could inhibit the maximum response to α,β‐meATP, but not shift the sensitivity of P2X3 receptors to α,β‐meATP.

To investigate whether the inhibition of ATP currents by DEX depended on membrane potentials, we observed the inhibitory effect of DEX on α,β‐meATP‐activated currents recorded at different clamping potentials. Figure 2C shows that DEX pretreatment (3 μM for 5min) inhibited the peak amplitudes of the three I_ATP,_ which were evoked by 100 μM α,β‐meATP when the membrane potential was clamped at −80 mV, −40 mV, and +20 mV, separately. Figure 2D shows the current‐voltage (I–V) curves for α,β‐meATP with and without DEX pretreatment. DEX did not change the reversal potential (near 0 mV) of the I‐V curve, but decreased the slope of curve. There was no significant difference in the DEX‐induced inhibition of ATP currents at different clamping potentials from −80 to 20 mV (*p* > 0.1, Bonferroni's post hoc test). The results indicated that DEX voltage independently inhibited P2X3 receptor‐mediated ATP currents.

### 3.3 DEX inhibits ATP currents via an α_2_‐ARs, G_i/o_ proteins and cAMP signaling pathway

**FIGURE 3:**
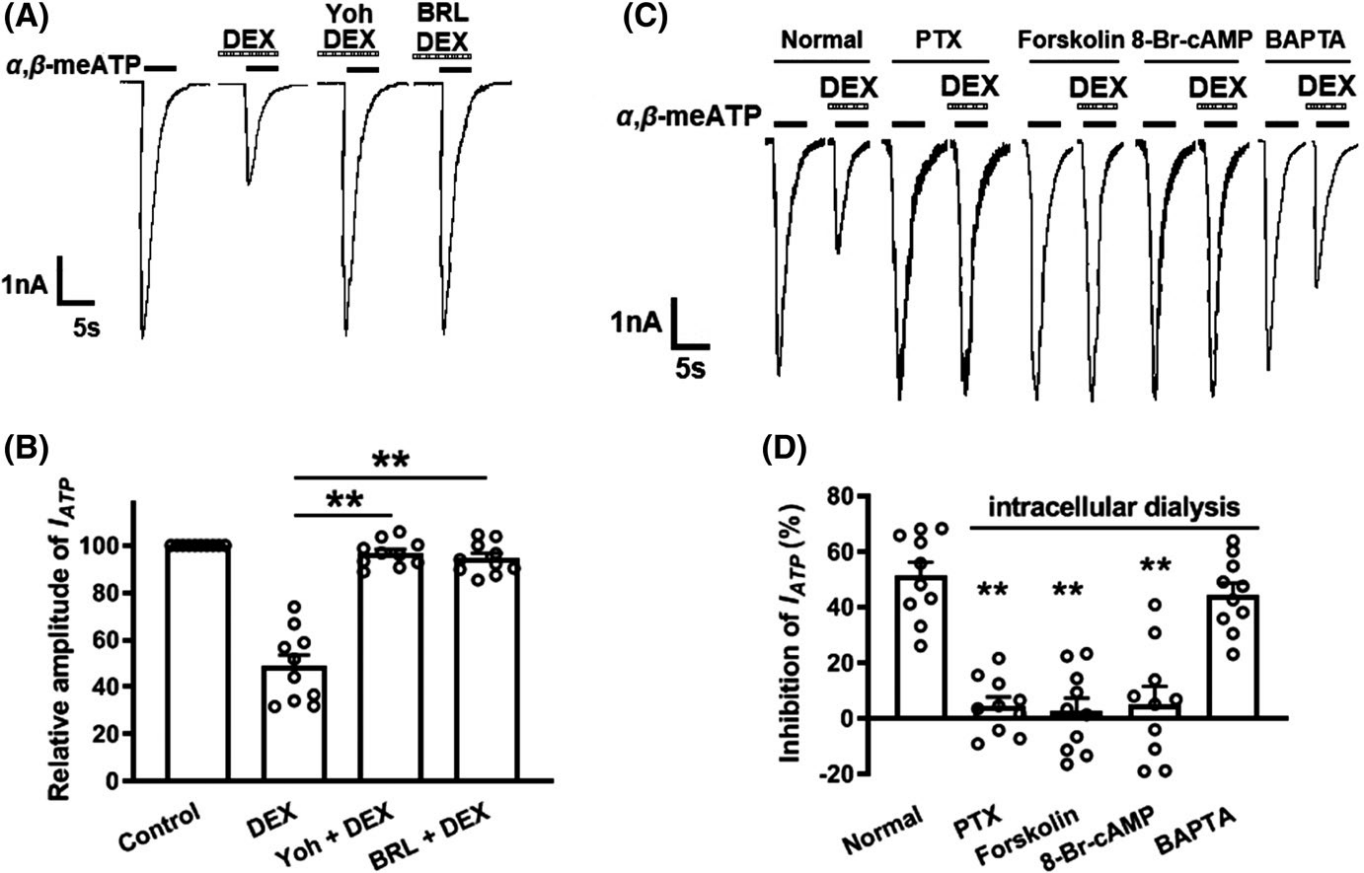
Participation of α_2A_‐ARs, G_i_ proteins, and cAMP signaling in the DEX‐induced inhibition of ATP currents. Representative current traces in (A) and the bar graph in (B) showed I_ATP_ were inhibited by DEX (3 μM) pre‐applied alone for 5 min in DRG cells, and the DEX‐induced suppression of I_ATP_ was blocked by the co‐application of the α_2_‐AR antagonist yohimbine (3 μM) or the α_2A_‐AR antagonist BRL44408 (3 μM). Currents in (B) were normalized to the control (100%). **p < 0.01, Bonferroni's post hoc test, compared with DEX column. n = 10 in each column. The current traces in (C) and the bar graph in (D) showed DEX (3 μM) had little effect on I_ATP_ in recording pipettes filled with PTX (1 μg/ml), forskolin (0.1 μM), or 8‐Br‐cAMP (1 mM) containing internal solution conditions, which was different from the inhibitory effect under normal internal solution conditions. However, intracellular application of BAPTA (10mM, a chelator of calcium ions) was unable to reverse the inhibitory effect of DEX on I_ATP_. **p < 0.01, Bonferroni's post hoc test, compared with normal column. n = 10 in each column

As a selective α_2_‐AR agonist, is the inhibitory effect of DEX on ATP currents mediated by α_2_‐ARs? We examined the effects of the α_2_‐AR antagonist yohimbine and the α_2A_‐AR antagonist BRL44408 on suppression of ATP currents by DEX. As shown in Figure 3A and B, unlike a decrease of 51.32 ± 4.63% in the I_ATP_ amplitude after 3 μM DEX pretreatment alone, the I_ATP_ amplitude decreased only 3.33 ± 1.24% and 3.72 ± 1.93 when 3 μM DEX was co‐treated with 3 μM yohimbine or 3 μM BRL44408. (*p* < 0.01, compared with DEX pretreatment alone, one‐way ANOVA followed by post hoc Bonferroni's test, n = 10). In other words, yohimbine and BRL44408 significantly blocked the inhibition of DEX on I_ATP_. The results indicated that DEX inhibited ATP currents through α_2A_‐ARs in DRG neurons.

α_2A_‐AR is coupled with G_i/o_ member of the G protein family, through which it can inhibit adenylyl cyclase and attenuate intracellular cAMP levels.^32^, ^33^ First, we explored whether G_i/o_ proteins contribute to the DEX‐induced suppression of ATP currents. A G_i/o_ protein inhibitor, pertussis toxin (PTX, 1 μg/mL), was applied internally to DRG neurons before DEX treatment. Figure 3C and D shows that 3 μM DEX failed to inhibit I_ATP_ in PTX‐treated cells, suggesting the DEX‐induced inhibition was significantly prevented by PTX. Second, we further explore intracellular signal transduction mechanisms underlying the DEX‐induced suppression. The adenylate cyclase activator forskolin or the cAMP analog 8‐Br‐cAMP was applied internally to DRG cells through recording patch pipettes. Unlike a significant decrease in I_ATP_ amplitude under the normal internal solution conditions, 3 μM DEX produced only decreases of 2.65 ± 5.58% and 5.21 ± 8.03% on I_ATP_, separately, after forskolin (0.1 μM) or 8‐Br‐cAMP (1 mM) was applied internally to DRG cells (*p* < 0.01, compared with normal internal solution, one‐way ANOVA followed by post hoc Bonferroni's test, n = 10; Figure 3C and D). In contrast, intracellular application of BAPTA (10 mM, a chelator of calcium ions) was unable to reverse the inhibitory effect of DEX on I_ATP_. These results suggested that DEX inhibited ATP currents via a G_i/o_ proteins and intracellular cAMP signaling pathway.

### 3.4 DEX suppresses α,β‐meATP‐evoked action potentials in rat DRG neurons

**FIGURE 4:**
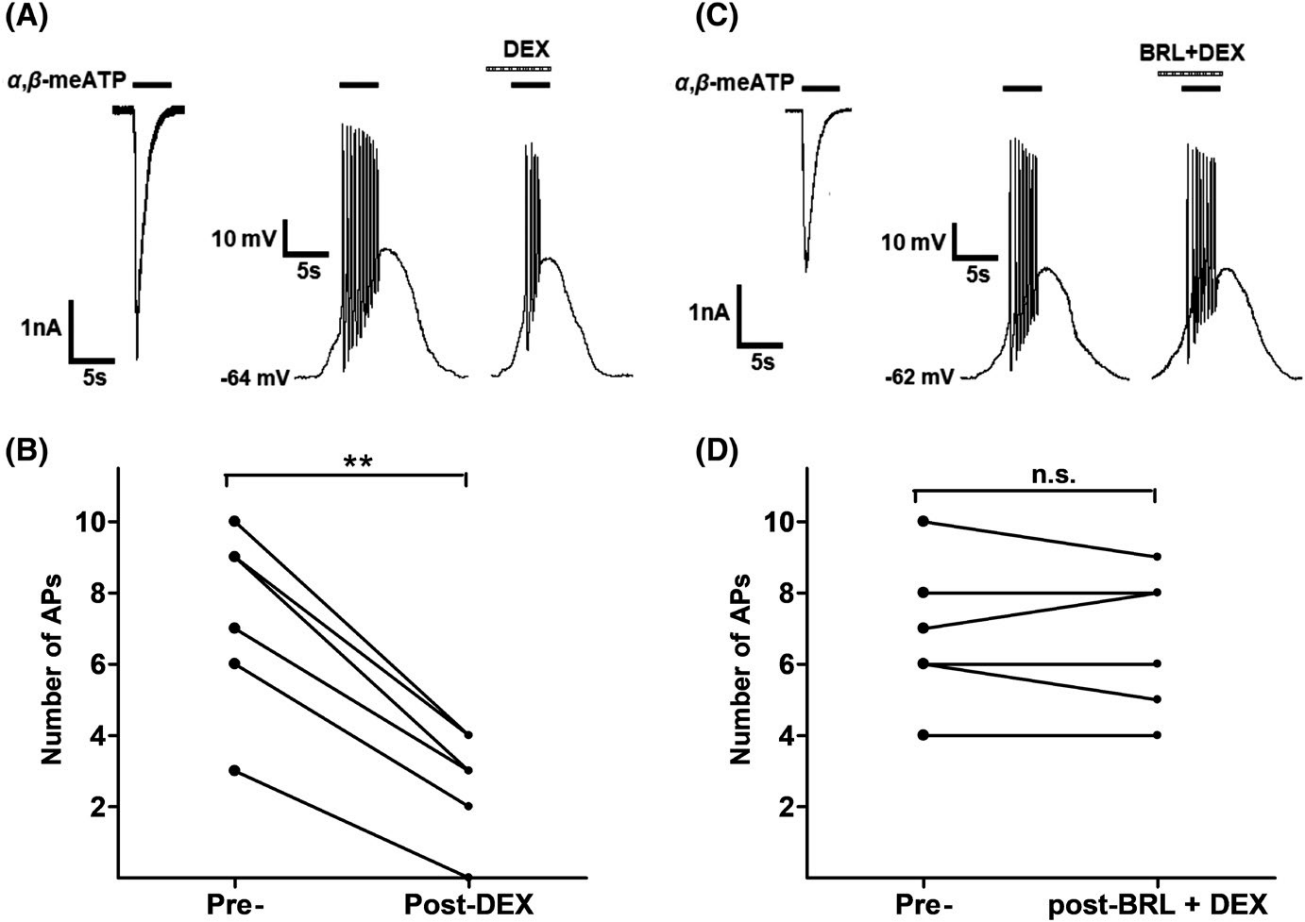
DEX‐induced suppression of α,β‐meATP‐evoked action potentials in rat DRG neurons. Original traces in (A) and (C) showed application of 100 μM α,β‐meATP to the same DRG cell caused an inward current and action potentials (APs) under voltage‐clamp and current‐clamp conditions, respectively. Original APs were recorded before and after application of DEX (3 μM for 5 min) alone (A) or co‐application of both DEX (3 μM) and BRL44408 (BRL, 3 μM) (C) in two different DRG neurons. Data in (B) and (D) showed application of DEX alone, but not co‐application of both DEX and BRL44408, significantly decreased the number of APs evoked by 100 μM α,β‐meATP. ** *p* < 0.01, paired *t* test, n = 6 cells

We further investigated the effect of DEX on action potentials (APs) triggered by α,β‐meATP. In the same DRG cell, 100 μM α,β‐meATP not only induced a large inward current under voltage‐clamp conditions but also evoked burst of APs under current‐clamp conditions (Figure 4A and C). Consistent with the results observed under voltage‐clamp conditions, DEX also exerted an inhibitory effect on α,β‐meATP‐evoked APs. As shown in Figure 4B, DEX (3 μM for 5 min) pretreatment significantly decreased the number of APs triggered by α,β‐meATP in six DRG cells (*p* < 0.01, paired t test). However, co‐application of 3 μM BRL44408 and 3 μM DEX failed to decrease the number of APs in other six DRG cells (*p *˃ 0.1, paired t test; Figure 4D). The results suggested that DEX also suppressed α,β‐meATP‐evoked APs through α_2A_‐ARs in rat DRG neurons.

### 3.5 DEX relieves α,β‐meATP‐induced nociceptive behaviors in rats

**FIGURE 5:**
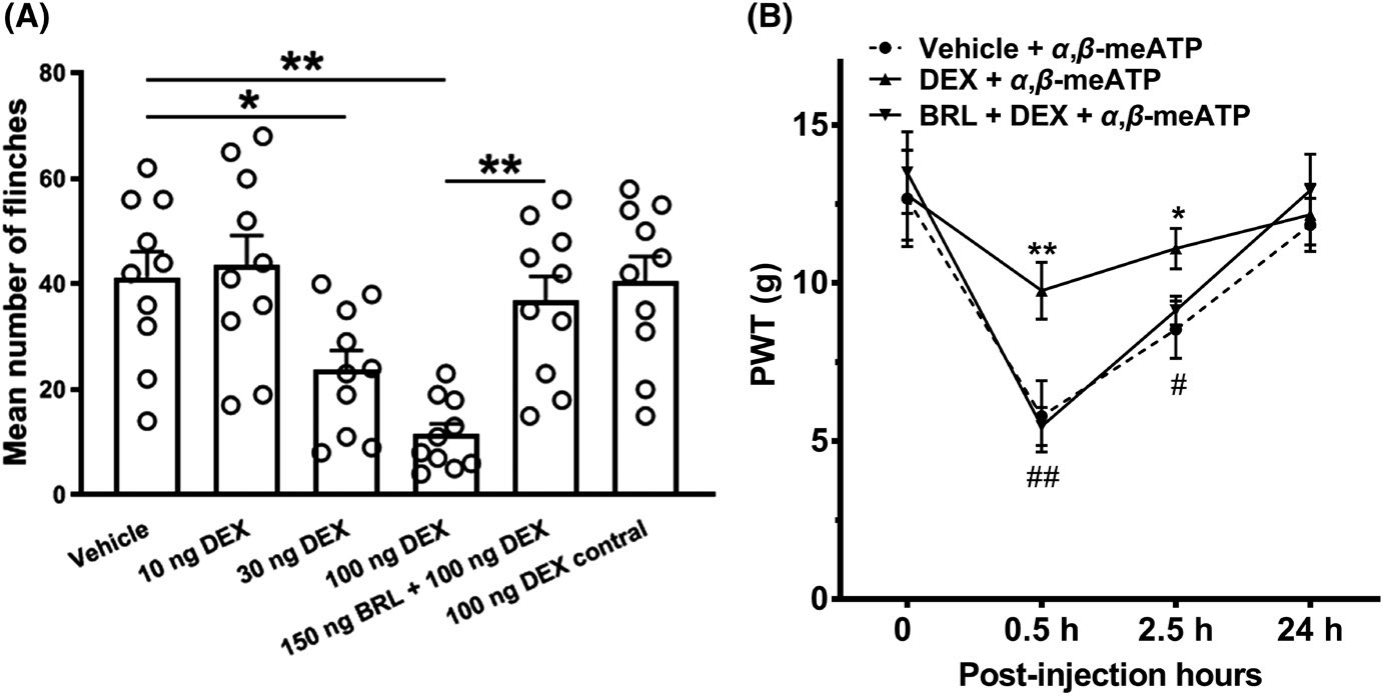
Relief of α,β‐meATP‐evoked nociceptive behaviors by DEX in rats. (A) Intraplantar injection of α,β‐meATP (50μg in 50μl) caused spontaneous flinching behaviors in rats. Intraplantar pretreatment of DEX (10, 30, and 100 ng) dose dependently decreased the number of α,β‐meATP‐induced flinching behaviors. The anti‐nociceptive effect of DEX (100ng) on the flinching behaviors was completely prevented by co‐treatment of the α_2A_‐AR antagonist BRL44408 (BRL, 150 ng). DEX (100 ng) had no effect on α,β‐meATP‐induced flinching behaviors when it was injected into the contralateral hindpaw and α,β‐meATP was injected into one hindpaw (100 ng contral). Bonferroni's post hoc test, *p < 0.05, **p < 0.01, compared with vehicle column; ## *p* < 0.01, compared with 100 ng DEX column. Each column represents the mean ±S.E.M. of 10 rats. (B) Intraplantar injection of α,β‐meATP (50 μg in 50 μl) also caused a remarkable decrease in paw withdrawal thresholds (PWT, in g) at 0.5 and 2.5 h after injection and recovery at 24 h. The α,β‐meATP‐induced mechanical allodynia was significantly relieved by intraplantar pretreatment of DEX (100 ng), but not co‐treatment of DEX (100 ng) and BRL44408 (BRL, 150 ng). *p < 0.05, **p <  0.01, Bonferroni's post hoc test, n = 10 rats in each group

Finally, we investigated whether the suppression of P2X3 receptors by DEX *in vitro* played a role in the nociceptive behaviors induced by α,β‐meATP *in vivo*. Figure 5A shows intraplantar injection of α,β‐meATP (50 μg in 50 μl) caused an intense spontaneous flinch/shaking response in rats, which was attenuated by intraplantar pretreatment of DEX. DEX dose dependently (10, 30, and 100 ng) relieved the nociceptive behaviors induced by α,β‐meATP (*p* < 0.05 and 0.01, one‐way ANOVA followed by post hoc Bonferroni's test, n = 10; Figure 5A). The anti‐nociceptive effect of 100 ng DEX was blocked by co‐treated 150 ng BRL44408 (*p* < 0.01, one‐way ANOVA followed by post hoc Bonferroni's test, n = 10; Figure 5A). In addition, 100 ng DEX had no effect on the α,β‐meATP‐induced nociceptive behaviors when injected into the contralateral paws. The results suggested that DEX had an anti‐nociceptive effect on the α,β‐meATP‐induced nociceptive behaviors *in vivo* through peripheral α_2A_‐ARs.

We also observe that the effect of DEX on the mechanical allodynia induced by α,β‐meATP in rats. Figure 5B shows intraplantar injection of α,β‐meATP (50 μg in 50 μl) resulted in a significant decrease in the paw withdrawal threshold (PWT) within 0.5 and 4 h after injection, and recovery at 24 h. Intraplantar pretreatment of DEX had also an analgesic effect on the mechanical allodynia. The α,β‐meATP‐induced mechanical allodynia significantly decreased within 0.5 and 4 h after 100 ng DEX pretreatment (*p* < 0.05 and 0.01, Bonferroni's post hoc test, compared with vehicle +α,β‐meATP group, n =  10 rats; Figure 5B). The analgesic effect of DEX was completely blocked by co‐treated 150 ng BRL44408 (*p* < 0.05 and 0.01, Bonferroni's post hoc test, compared with DEX +α,β‐meATP group, n =  10 rats; Figure 5B). The results suggested that DEX had also an analgesic effect on the α,β‐meATP‐induced mechanical allodynia through peripheral α_2A_‐ARs.

## 4 DISCUSSION

The present data demonstrated that the selective α_2_‐AR agonist DEX suppressed the electrophysiological activity of P2X3 receptors in rat DRG neurons. DEX reduced not only the amplitude of ATP currents but also the action potential bursts induced by α,β‐meATP. The α_2A_‐ARs, PTX‐sensitive G_i/o_ proteins, and cAMP signaling cascades were involved in the inhibition of P2X3 receptors by DEX. Behaviorally, DEX also relieved P2X3 receptor‐mediated nociceptive behaviors in rats by activating peripheral α_2A_‐ARs.

The recorded ATP currents in the present experiments were mediated by P2X3 receptors, because they could be blocked by specifical antagonist of P2X3 and P2X2/3 receptor A‐317491.^21^ Moreover, α,β‐meATP can only activate P2X3 and P2X1 receptors.^31^ ATP receptors include P2X1‐7 subtypes. Among all subtypes, P2X3 receptor subtype is mainly located in a subset of small‐ and medium‐sized nociceptive DRG neurons.^17^, ^18^, ^19^ The present study showed that DEX, a selective α_2A_‐AR agonist, concentration dependently inhibited P2X3 receptor‐mediated ATP currents. The DEX‐induced suppression did not alter the sensitivity of P2X3 receptor to α,β‐meATP, but decreased the maximum response to α,β‐meATP. DEX suppressed ATP currents in voltage‐independent manner. P2X3 receptor is a cation‐permeable channel. The activation of P2X3 receptors by α,β‐meATP evokes a rapid inward current sufficient to induce membrane depolarization and to generate firing.^31^, ^34^ Under the current‐clamp conditions, DEX also suppressed the number of APs evoked by α,β‐meATP in rat DRG neurons. Obviously, the two results corroborated each other in the current‐clamp and voltage‐clamp experiments.

DEX suppressed ATP currents through α_2A_‐ARs, since DEX‐induced inhibition was mimicked by another α_2_‐AR agonist clonidine and completely blocked by the α_2A_‐AR antagonist BRL44408. All three subtypes of α_2A_‐, α_2B_‐, and α_2C_‐AR have been identified in DRGs.^4^ Subtypes of α_2A_‐ and α_2C_‐AR are expressed in most DRG neurons, while is found only in a very small population.^3^, ^4^, ^35^ In addition, both DEX and clonidine have more specific activity against α_2A_‐AR subtype.^36^ Considering that the α_2A_‐ARs maybe co‐express with P2X3 receptors in DRG neurons, it was completely possible that DEX decreased ATP currents through α_2A_‐ARs. The present study showed that the inhibition of ATP currents by DEX / clonidine only occurred in some but not all DRG neurons, which may be related to the degree of the co‐existence of α_2A_‐ARs and P2X3 receptors in rat DRG neurons, although the evidence of morphological co‐existence remains to be elucidated. It has been shown that the activation of α_2A_‐AR subtypes also modulates the functional activity of other cation channels, such as Nav1.8, TRPV1, TRPM8, and ASICs in DRG neurons.^14^, ^16^, ^29^, ^37^

α_2A_‐AR belongs to G_i/o_ protein‐coupled receptor family that can inhibit adenylyl cyclase and attenuate intracellular cAMP levels.^32^, ^33^ The present data showed that G_i/o_ proteins and intracellular cAMP signaling were involved in the DEX‐induced suppression of ATP currents, as demonstrated by the results that the DEX‐induced inhibition was lack after DRG neurons were intracellularly dialyzed with the G_i/o_ protein inhibitor PTX, the adenylate cyclase activator forskolin, or the cAMP analog 8‐Br‐cAMP. Previous studies have showed that P2X3 receptor‐mediated ATP currents are regulated by intracellular cAMP/PKA signaling. Ma group reported that 17β‐estradiol and progesterone rapidly attenuate ATP currents in DRG neurons via a cAMP‐PKA signaling pathway.^38^, ^39^ On contrary, PGE2 enhances P2X3 receptor‐mediated currents in DRG neurons via its EP3 receptor and a cAMP/PKA‐dependent signaling pathway.^40^ It has been also reported that PKC and PKA potentiate α,β‐meATP‐induced currents in rat DRG neurons in a synergistic manner.^41^ Obviously, the latter two reported studies are consistent with the current results that the activation of α_2A_‐ARs by DEX reduced ATP currents through down‐regulating intracellular cAMP signaling pathway. Similarly, cannabinoid inhibits ATP‐induced currents and calcium influx through G_i/o_ protein‐coupled CB1 cannabinoid receptors and AC‐cAMP‐PKA signaling pathway in rat trigeminal ganglionic neurons and cultured spinal dorsal horn neurons.^42^, ^43^ In addition, intracellular application of BAPTA, a chelator of calcium ions, failed to reverse the inhibitory effect of DEX on α,β‐meATP‐induced currents, suggesting no involvement of intracellular Ca.^2^ ^+^

P2X3 receptors are not only expressed in the somata of DRG neurons but also expressed in peripheral nociceptive sensory nerve endings. Injection of ATP into the skin elicits pain via P2X3 receptors.^44^ Intraplantar injection of P2X3 receptor agonists results in spontaneous pain behaviors and mechanical allodynia in rats.^25^, ^45^, ^46^ Local pretreatment of α_1_‐AR agonists augment P2X3 receptor‐mediated flinching behaviors in rats.^47^ This behavioral finding is consistent with electrophysiological results that the activation of α1‐ARs by noradrenaline potentiates ATP‐evoked currents in DRG neurons by activating PKC.^26^ The present results showed that peripheral pretreatment of the DEX dose dependently relieved the α,β‐meATP‐induced nociceptive behaviors. The anti‐nociceptive effect of DEX occurred locally rather than systematically by directly activating peripheral α_2A_‐ARs localized on nociceptors. First, treatment of contralateral hindpaws with DEX failed to relieve the α,β‐meATP‐induced nociceptive behaviors. Second, the anti‐nociceptive effect of DEX was abolished by local treatment with BRL44408, an α_2A_‐AR antagonist. In further mechanical allodynia experiments, DEX had also an analgesic effect on the α,β‐meATP‐induced mechanical allodynia through peripheral α_2A_‐ARs. The present behavioral results apparently confirmed the aforementioned electrophysiological data and vice versa. Somata of DRG neurons in the electrophysiological experiments was used as a model to observe the characteristics of peripheral terminals. It may occur that the activation of α_2A_‐ARs by DEX suppressed co‐existed P2X3 receptors in peripheral nociceptive sensory nerve endings, which could reduce P2X3 receptor‐mediated currents and action potential bursts, and then result in attenuated nociceptive behaviors.

## 5 CONCLUSIONS

Under inflammatory and neuropathic pain conditions, not only a large amount of ATP is released from damaged cells but also total expression and membrane expression of P2X3 receptors increases in DRG neurons.^25^, ^48^, ^49^, ^50^ These P2X3 receptors, activated by released ATP, plays a prominent role in some pain states.^51^ Our results suggested that DEX suppressed P2X3 receptor‐mediated the electrophysiological activity and nociception through α_2A_‐ARs, revealing a peripheral novel mechanism underlying the analgesia of DEX. Clinically, DEX, even when applied locally, may also effectively relieve pain involving peripheral P2X3 receptors.

## CONFLICT OF INTEREST

All authors declare no conflicts of interest.

## AUTHOR CONTRIBUTIONS

WPH designed this research. JWH, WLQ, QL, SW, TTL, and CYQ performed the experiments. JWH, WLQ, and QL participated in data analysis. JWH, WLQ, QL, and WPH wrote the paper. All authors contributed substantially to this research and reviewed this manuscript.

## ETHICAL APPROVAL

The animal study was reviewed and approved by the animal research ethics committee of Hubei University of Science and Technology.

## Data Availability

All data generated or used during the study appear in the submitted article.

## References

[1] Bylund DB , Eikenberg DC , Hieble JP , et al. International union of pharmacology nomenclature of adrenoceptors. Pharmacol Rev. 1994;46(2):121‐136. 7938162

[2] Pertovaara A . Noradrenergic pain modulation. Prog Neurobiol. 2006;80(2):53‐83. 17030082 10.1016/j.pneurobio.2006.08.001

[3] Pertovaara A . The noradrenergic pain regulation system: a potential target for pain therapy. Eur J Pharmacol. 2013;716(1–3):2‐7. 23500194 10.1016/j.ejphar.2013.01.067

[4] Shi TS , Winzer‐Serhan U , Leslie F , Hokfelt T . Distribution and regulation of alpha(2)‐adrenoceptors in rat dorsal root ganglia. Pain. 2000;84(2–3):319‐330. 10666537 10.1016/s0304-3959(99)00224-9

[5] Stone LS , Broberger C , Vulchanova L , et al. Differential distribution of alpha2A and alpha2C adrenergic receptor immunoreactivity in the rat spinal cord. J Neurosci. 1998;18(15):5928‐5937. 9671679 10.1523/JNEUROSCI.18-15-05928.1998 PMC6793037

[6] Nicholson R , Dixon AK , Spanswick D , Lee K . Noradrenergic receptor mRNA expression in adult rat superficial dorsal horn and dorsal root ganglion neurons. Neurosci Lett. 2005;380(3):316‐321. 15862909 10.1016/j.neulet.2005.01.079

[7] Gold MS , Dastmalchi S , Levine JD . Alpha 2‐adrenergic receptor subtypes in rat dorsal root and superior cervical ganglion neurons. Pain. 1997;69(1–2):179‐190. 9060029 10.1016/s0304-3959(96)03218-6

[8] Aho M , Lehtinen AM , Erkola O , Kallio A , Korttila K . The effect of intravenously administered dexmedetomidine on perioperative hemodynamics and isoflurane requirements in patients undergoing abdominal hysterectomy. Anesthesiology. 1991;74(6):997‐1002. 1675042 10.1097/00000542-199106000-00005

[9] Kimura M , Saito S , Obata H . Dexmedetomidine decreases hyperalgesia in neuropathic pain by increasing acetylcholine in the spinal cord. Neurosci Lett. 2012;529(1):70‐74. 22917606 10.1016/j.neulet.2012.08.008

[10] Zhang X , Bai X . New therapeutic uses for an alpha2 adrenergic receptor agonist–dexmedetomidine in pain management. Neurosci Lett. 2014;561:7‐12. 24373989 10.1016/j.neulet.2013.12.039

[11] Kamibayashi T , Maze M . Clinical uses of alpha2 ‐adrenergic agonists. Anesthesiology. 2000;93(5):1345‐1349. 11046225 10.1097/00000542-200011000-00030

[12] Poree LR , Guo TZ , Kingery WS , Maze M . The analgesic potency of dexmedetomidine is enhanced after nerve injury: a possible role for peripheral alpha2‐adrenoceptors. Anesth Analg. 1998;87(4):941‐948. 9768799 10.1097/00000539-199810000-00037

[13] Lee HG , Choi JI , Kim YO , Yoon MH . The role of alpha‐2 adrenoceptor subtype in the antiallodynic effect of intraplantar dexmedetomidine in a rat spinal nerve ligation model. Neurosci Lett. 2013;557(Pt 8):118‐122. 24161890 10.1016/j.neulet.2013.10.002

[14] Gu XY , Liu BL , Zang KK , et al. Dexmedetomidine inhibits Tetrodotoxin‐resistant Nav1.8 sodium channel activity through Gi/o‐dependent pathway in rat dorsal root ganglion neurons. *Mol* . Brain. 2015;8:15. 10.1186/s13041-015-0105-2 PMC4350947 25761941

[15] Im ST , Jo YY , Han G , Jo HJ , Kim YH , Park CK . Dexmedetomidine inhibits voltage‐gated sodium channels via alpha2‐adrenoceptors in trigeminal ganglion neurons. Mediators Inflamm. 2018;2018:1782719. 30245586 10.1155/2018/1782719 PMC6139198

[16] Lee BM , Jang Y , Park G , et al. Dexmedetomidine modulates transient receptor potential vanilloid subtype 1. Biochem Biophys Res Commun. 2020;522(4):832‐837. 31796207 10.1016/j.bbrc.2019.11.146

[17] Bradbury EJ , Burnstock G , McMahon SB . The expression of P2X3 purinoreceptors in sensory neurons: effects of axotomy and glial‐derived neurotrophic factor. Mol Cell Neurosci. 1998;12(4–5):256‐268. 9828090 10.1006/mcne.1998.0719

[18] Dunn PM , Zhong Y , Burnstock G . P2X receptors in peripheral neurons. Prog Neurobiol. 2001;65(2):107‐134. 11403876 10.1016/s0301-0082(01)00005-3

[19] Vulchanova L , Riedl MS , Shuster SJ , et al. Immunohistochemical study of the P2X2 and P2X3 receptor subunits in rat and monkey sensory neurons and their central terminals. Neuropharmacology. 1997;36(9):1229‐1242. 9364478 10.1016/s0028-3908(97)00126-3

[20] Honore P , Kage K , Mikusa J , et al. Analgesic profile of intrathecal P2X(3) antisense oligonucleotide treatment in chronic inflammatory and neuropathic pain states in rats. Pain. 2002;99(1–2):11‐19. 12237180 10.1016/s0304-3959(02)00032-5

[21] Jarvis MF , Burgard EC , McGaraughty S , et al. A‐317491, a novel potent and selective non‐nucleotide antagonist of P2X3 and P2X2/3 receptors, reduces chronic inflammatory and neuropathic pain in the rat. Proc Natl Acad Sci U S A. 2002;99(26):17179‐17184. 12482951 10.1073/pnas.252537299 PMC139289

[22] Hamilton SG , McMahon SB , Lewin GR . Selective activation of nociceptors by P2X receptor agonists in normal and inflamed rat skin. J Physiol. 2001;534(Pt. 2):437‐445. 11454962 10.1111/j.1469-7793.2001.00437.x PMC2278707

[23] Sharp CJ , Reeve AJ , Collins SD , et al. Investigation into the role of P2X(3)/P2X(2/3) receptors in neuropathic pain following chronic constriction injury in the rat: an electrophysiological study. Br J Pharmacol. 2006;148(6):845‐852. 16770326 10.1038/sj.bjp.0706790 PMC1617075

[24] McGaraughty S , Wismer CT , Zhu CZ , et al. Effects of A‐317491, a novel and selective P2X3/P2X2/3 receptor antagonist, on neuropathic, inflammatory and chemogenic nociception following intrathecal and intraplantar administration. Br J Pharmacol. 2003;140(8):1381‐1388. 14623769 10.1038/sj.bjp.0705574 PMC1574160

[25] Xiang Z , Xiong Y , Yan N , et al. Functional up‐regulation of P2X 3 receptors in the chronically compressed dorsal root ganglion. Pain. 2008;140(1):23‐34. 18715715 10.1016/j.pain.2008.07.006 PMC2667225

[26] Maruo K , Yamamoto H , Yamamoto S , et al. Modulation of P2X receptors via adrenergic pathways in rat dorsal root ganglion neurons after sciatic nerve injury. Pain. 2006;120(1–2):106‐112. 16360272 10.1016/j.pain.2005.10.016

[27] Jin Y , Wei S , Liu TT , Qiu CY , Hu WP . Acute P38‐mediated enhancement of P2X3 receptor currents by TNF‐alpha in rat dorsal root ganglion neurons. J Inflamm Res. 2021;14:2841‐2850. 34234509 10.2147/JIR.S315774 PMC8254564

[28] Jin Y , Qiu CY , Wei S , Han L , Liu TT , Hu WP . Potentiation of P2X3 receptor mediated currents by endothelin‐1 in rat dorsal root ganglion neurons. Neuropharmacology. 2020;181:108356. 33069757 10.1016/j.neuropharm.2020.108356

[29] Wei S , Qiu CY , Jin Y , Liu TT , Hu WP . Dexmedetomidine inhibits ASIC activity via activation of alpha2A adrenergic receptors in rat dorsal root ganglion neurons. Front Pharmacol. 2021;12: 685460. 34108881 10.3389/fphar.2021.685460 PMC8181722

[30] Chaplan SR , Bach FW , Pogrel JW , Chung JM , Yaksh TL . Quantitative assessment of tactile allodynia in the rat paw. J Neurosci Methods. 1994;53(1):55‐63. 7990513 10.1016/0165-0270(94)90144-9

[31] North RA . Molecular physiology of P2X receptors. Physiol Rev. 2002;82(4):1013‐1067. 12270951 10.1152/physrev.00015.2002

[32] Wu YY , Goldfien A , Roberts JM . Alpha adrenergic stimulation reduces cyclic adenosine 3’,5'‐monophosphate generation in rabbit myometrium by two mechanisms. Biol Reprod. 1988;39(1):58‐65. 2462919 10.1095/biolreprod39.1.58

[33] Cotecchia S , Stanasila L , Diviani D . Protein‐protein interactions at the adrenergic receptors. Curr Drug Targets. 2012;13(1):15‐27. 21777184 10.2174/138945012798868489 PMC3290771

[34] Xu J , Chu KL , Brederson JD , Jarvis MF , McGaraughty S . Spontaneous firing and evoked responses of spinal nociceptive neurons are attenuated by blockade of P2X3 and P2X2/3 receptors in inflamed rats. J Neurosci Res. 2012;90(8):1597‐1606. 22422599 10.1002/jnr.23042

[35] Chung K , Kim HJ , Na HS , Park MJ , Chung JM . Abnormalities of sympathetic innervation in the area of an injured peripheral nerve in a rat model of neuropathic pain. Neurosci Lett. 1993;162(1–2):85‐88. 7907173 10.1016/0304-3940(93)90566-4

[36] Noyer M , de Laveleye F , Vauquelin G , Gobert J , Wulfert E . Mivazerol, a novel compound with high specificity for alpha 2 adrenergic receptors: binding studies on different human and rat membrane preparations. Neurochem Int. 1994;24(3):221‐229. 8025531 10.1016/0197-0186(94)90079-5

[37] Bavencoffe A , Gkika D , Kondratskyi A , et al. The transient receptor potential channel TRPM8 is inhibited via the alpha 2A adrenoreceptor signaling pathway. J Biol Chem. 2010;285(13):9410‐9419. 20110357 10.1074/jbc.M109.069377 PMC2843190

[38] Lu Y , Jiang Q , Yu L , et al. 17beta‐estradiol rapidly attenuates P2X3 receptor‐mediated peripheral pain signal transduction via ERalpha and GPR30. Endocrinology. 2013;154(7):2421‐2433. 23610132 10.1210/en.2012-2119

[39] Fan J , Lu Y , Yu LH , et al. Progesterone rapidly attenuates ATP‐evoked transient currents in cultured rat dorsal root ganglion neurons. Pharmacology. 2011;87(1–2):36‐44. 21178388 10.1159/000322535

[40] Wang C , Li GW , Huang LY . Prostaglandin E2 potentiation of P2X3 receptor mediated currents in dorsal root ganglion neurons. Mol Pain. 2007;3:22. 17692121 10.1186/1744-8069-3-22 PMC2063498

[41] Wang S , Dai Y , Kobayashi K , et al. Potentiation of the P2X3 ATP receptor by PAR‐2 in rat dorsal root ganglia neurons, through protein kinase‐dependent mechanisms, contributes to inflammatory pain. Eur J Neurosci. 2012;36(3):2293‐2301. 22616675 10.1111/j.1460-9568.2012.08142.x

[42] Shen JJ , Liu CJ , Li A , et al. Cannabinoids inhibit ATP‐activated currents in rat trigeminal ganglionic neurons. Sheng Li Xue Bao. 2007;59(6):745‐752. 18157466

[43] Long J , Lei X , Chen M , et al. CB1 receptors mediated inhibition of ATP‐induced [Ca(2+)]i increase in cultured rat spinal dorsal horn neurons. Neurochem Res. 2018;43(2):267‐275. 29127599 10.1007/s11064-017-2414-6

[44] Cockayne DA , Hamilton SG , Zhu QM , et al. Urinary bladder hyporeflexia and reduced pain‐related behaviour in P2X3‐deficient mice. Nature. 2000;407(6807):1011‐1015. 11069181 10.1038/35039519

[45] Gu Y , Li G , Chen Y , Huang LM . Epac‐protein kinase C alpha signaling in purinergic P2X3R‐mediated hyperalgesia after inflammation. Pain. 2016;157(7):1541‐1550. 26963850 10.1097/j.pain.0000000000000547 PMC4912236

[46] Hamilton SG , Wade A , McMahon SB . The effects of inflammation and inflammatory mediators on nociceptive behaviour induced by ATP analogues in the rat. Br J Pharmacol. 1999;126(1):326‐332. 10051152 10.1038/sj.bjp.0702258 PMC1565771

[47] Meisner JG , Waldron JB , Sawynok J . Alpha1‐adrenergic receptors augment P2X3 receptor‐mediated nociceptive responses in the uninjured state. J Pain. 2007;8(7):556‐562. 17512257 10.1016/j.jpain.2007.02.434

[48] Xu GY , Huang LY . Peripheral inflammation sensitizes P2X receptor‐mediated responses in rat dorsal root ganglion neurons. J Neurosci. 2002;22(1):93‐102. 11756492 10.1523/JNEUROSCI.22-01-00093.2002 PMC6757597

[49] Cook SP , McCleskey EW . Cell damage excites nociceptors through release of cytosolic ATP. Pain. 2002;95(1–2):41‐47. 11790466 10.1016/s0304-3959(01)00372-4

[50] Chen Y , Li GW , Wang C , Gu Y , Huang LM . Mechanisms underlying enhanced P2X receptor‐mediated responses in the neuropathic pain state. Pain. 2005;119(1–3):38‐48. 16298067 10.1016/j.pain.2005.09.007

[51] Inoue K , Tsuda M . Nociceptive signaling mediated by P2X3, P2X4 and P2X7 receptors. Biochem Pharmacol. 2021;187: 114309. 33130129 10.1016/j.bcp.2020.114309

